# Predictive value of women’s weight trajectories in determining familial cardiovascular disorders: a family-based longitudinal study

**DOI:** 10.1038/s41598-021-96154-5

**Published:** 2021-08-27

**Authors:** Parisa Naseri, Parisa Amiri, Amirali Zareie Shab-khaneh, Fereidoun Azizi

**Affiliations:** 1 Research Center for Social Determinants of Health, Research Institute for Endocrine Sciences, Shahid Beheshti University of Medical Sciences, P.O. Box 19395-4763, Tehran, Islamic Republic of Iran; 2 Department of Epidemiology and Biostatistics, School of Public Health, Tehran University of Medical Sciences, Tehran, Islamic Republic of Iran; 3 Endocrine Research Center, Research Institute for Endocrine Sciences, Shahid Beheshti University of Medical Sciences, Tehran, Islamic Republic of Iran

**Keywords:** Cardiovascular diseases, Cardiology, Health occupations, Risk factors

## Abstract

Considering the dynamic nature of body mass index (BMI) and its importance in determining cardiovascular risks, this study aimed to investigate the life-course trajectory pattern of women’s BMI and its association with cardiovascular risk factors. A total of 1356 couples with 2976 children were recruited and followed up for an average period of 20 years. Latent growth curve modeling was applied to determine women's BMI trajectories; logistic regression was used to investigate the associations between trajectory patterns and cardiovascular risk factors, including hypertension (HTN), dyslipidemia, diabetes mellitus (DM), and obesity. Women were classified into three trajectories, including normal, stage 1 obesity, and stage 2 obesity. Compared to women’s in the normal trajectory group, those in obesity trajectories had higher odds ratios for HTN, DM, and dyslipidemia. Men with obese spouses showed a higher rate of HTN 1.54 (95% CI 1.05–2.25) and DM 1.55; (95% CI 1.00–2.44). The odds of men’s obesity were higher in obese spouses (OR 1.70; 95% CI 1.10–2.62). Offspring of stage 2 obese (OR 2.39; 95% CI 1.67–3.44) and stage 1 obese (OR 4.81; 95% CI 3.16–7.34) mothers were more likely to be obese. Our findings emphasized paying more attention to women with excessive weight to promote familial cardiovascular health in the communities.

## Introduction

Cardiovascular diseases (CVDs) are the leading causes of death in developed and developing countries^1,2^. The worldwide mortality rate from CDVs showed a rising trend, up to 15%, between 2006 and 2016^1^. According to existing evidence, CVDs are also lead to 12% of global disability during life years^3^. In Iran, around 50% of deaths are caused by CVDs^4^. Obesity, diabetes mellitus (DM)^5^, dyslipidemia, and hypertension (HTN)^6^ as well-documented risk factors for CVDs, could significantly threaten individuals' health. It is predicted that by 2030, a total of 2.16 and 1.12 billion individuals will be overweight and obese, respectively, and 30% and 9.3% of adults worldwide have hypertension and DM, respectively^7–9^. In Iran, the rate of excessive weight is considerably high; recent data showed 12.8–76.4 and 2.4–35.4% of Iranian adults suffer from overweight and obesity, respectively^8^. Moreover, the prevalence of hypertension and DM were 29.9% and 9.94%, respectively^10,11^.

The familial environment has been proposed as an essential contributor to CVD risk factors, including obesity^12^. Socioeconomic status (SES) and parental modeling of eating behaviors are examples of familial factors that have been related to developing CVD risk factors^13–15^. A growing number of studies showed the association between parental obesity and their offsprings’ weight status^16–18^. Studies indicated parental body mass index (BMI), particularly mother’s BMI, as the primary determinant of excessive weight gain in children during the life span from childhood to adulthood. It is significantly contributed to other cardio-metabolic risk factors^19^. However, the relationships between mothers’ weight status and metabolic complications in offspring are under debate. This issue of whether offspring with obese mothers are at higher risk for cardiometabolic impairment needs to be determined^20^.

In addition to offspring, weight status in women may affect their spouses’ cardiovascular risk factors. A study assessed the reciprocal effects of couple obesity and indicated that excessive weight in wives increased the risk of type 2 diabetes in their husbands; however, this effect was not observed in women^21^. This finding indicates the potential role of women’s weight status in association with their husbands’ cardiovascular risk factors. The spousal concordance, defined as consistency in health status between a husband and a wife, has been well known^22^. Several cross-sectional studies on spousal concordance have been conducted in chronic diseases, including hypertension^23,24^, cardiovascular diseases^25,26^, cancers, and type 2 diabetes^27^, which may be explained by the shared environment^22^. In Iran, the spousal concordance for type 2 diabetes and hypertension has been previously shown in cross-sectional studies^28,29^.

Several studies investigated the cross-sectional association between BMI and CVD risk factors^30^. Due to the changes in BMI patterns in recent years, the growth trajectories have been used to assess the life-course prevention of CVDs. A few studies are available on the long-term life-course change of BMI; however, no findings are revealed on the cardiovascular risk factors associated with the life-long change of women’s BMI in familial association^28,31–33^. In addition, one cannot extend these findings due to the different socio-environmental factors among populations. To the best of our knowledge, this is the first longitudinal study in the world that aimed to determine the life-course trajectory patterns of women’s BMI and identify which trajectory pattern was associated with a higher cardiovascular risk in whole family members as well as women’s cardiovascular risk factors.

## Methods

### Study design and population

This study used data from the Tehran Lipid and Glucose Study (TLGS), an ongoing population-based cohort with every-three-year measurements to determine the prevalence of non-communicable diseases (NCDs) risk factors in a representative sample of residents of district no.13 of Tehran. The first survey, including 15,005 participants (women and men, aged ≥ three years), was initiated in 1999–2001. Details of the TLGS have previously been published^34^.

**Figure 1:**
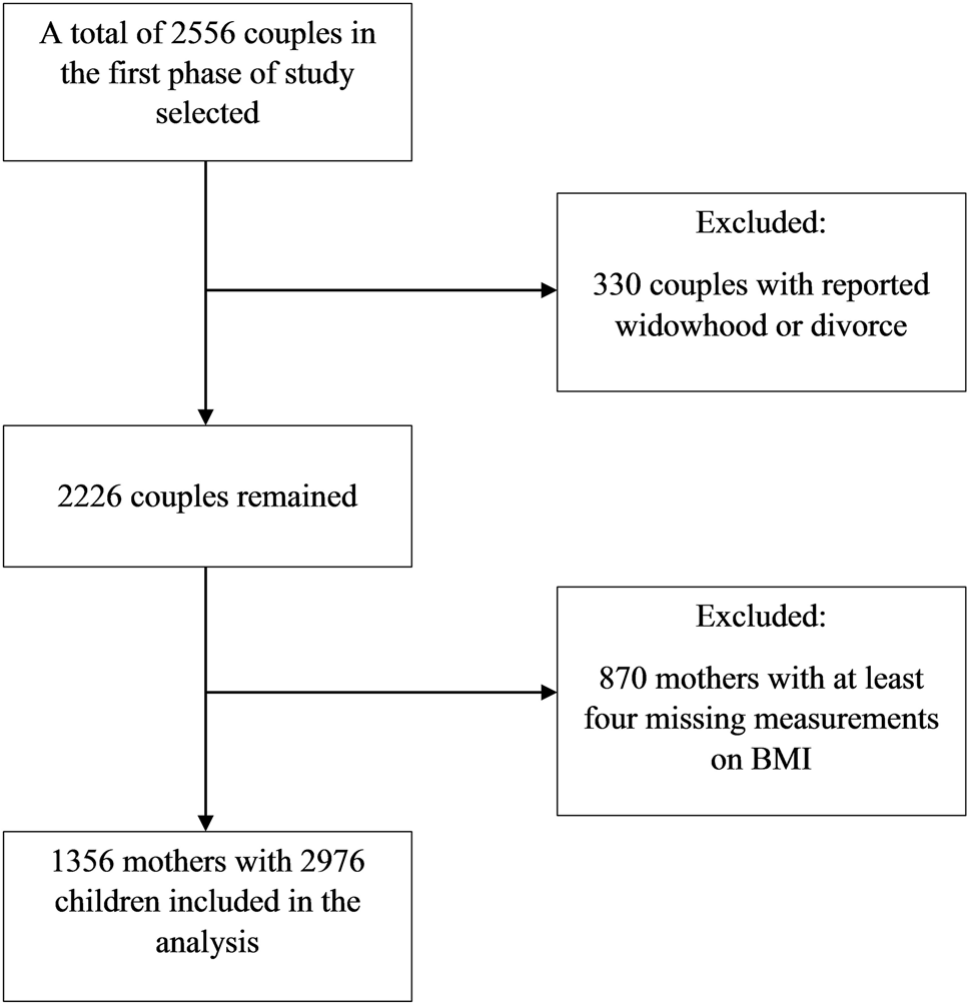
Flowchart of the study population.

For the present study, 2556 couples were selected from the first phase and followed for 20 years (till phase 6); after the exclusion of 330 couples with reported widowhood or divorce, 2226 couples remained. Afterward, mothers with at least four missing measurements on BMI were excluded (n = 870). The final analytic sample for latent class growth analysis (LCGA) comprises 1356 mothers with 2976 children (51.8% girls), aged ≤ 18 years at baseline. The study design was shown in Fig. 1. Written informed consent was obtained from all participants. The study protocol was approved by the ethics committee of the Research Institute for Endocrine Sciences (RIES) of the Shahid Beheshti University of Medical Sciences.

### Measurements

Trained interviewers collected data on age, sex, educational level, marital status, smoking status, physical activity, and medical and drug history using a pretested questionnaire. Blood pressure was recorded in a sitting position using a standard mercury sphygmomanometer with the cuff placed on the right arm. Two measurements were taken twice at the interval of 5 min each, and mean values were considered for systolic and diastolic blood pressure (SBP and DBP)^34^. Blood glucose and lipid levels, including triglycerides (TGs) and high-density lipoprotein (HDL-C), total cholesterol (TC), fasting plasma glucose (FPG), and 2‐hour post‐load plasma glucose (2 h‐PLPG), were measured using a morning blood sample which was obtained from participants in each phase at the TLGS research laboratory on the day of blood collection^34^. Weight of individuals was measured using digital scales, in light clothing and without shoes, and recorded to the nearest 100 g. Height was measured using a tape meter. BMI was calculated as weight in kilograms divided by square of height in meters. Reliable and validated the Modifiable Activity Questionnaire (MAQ) was used to measure three forms of activities, including leisure time, job, and household activities in the past year based on MET-minutes/week^35^.

### Definition of terms

Hypertension was defined as mean SBP ≥ 140 mm Hg or mean DBP ≥ 90 mm Hg or taking antihypertensive drugs^36^, and DM was defined as FPG ≥ 7 mmol/L or two hour‐PLPG ≥ 11.1 mmol/L^37^ or using glucose‐lowering medications. Dyslipidemia was defined as TG ≥ 1.69 mmol/L or HDL‐C levels < 1.04 mmol/L in men and < 1.29 mmol/L in women or TC ≥ 5.2 mmol/L or using lipid-lowering medications^38^. A BMI equal to or more than 30 kg/m^2^ in adults is defined as obesity^39^. Education level was classified into three categories: primary, secondary, and higher. Smoking status was classified as a smoker (current smokers) and nonsmoker (past or never smokers). A person who smokes cigarettes or uses other tobacco products daily or occasionally was considered as current smoker.

### Statistical analysis

Baseline characteristics of the mothers, fathers, and their offspring were summarized as means ± SD and frequencies (percentages) for continuous and categorical variables, respectively. Results were stratified based on mother trajectories. Continuous and categorical variables were compared between mother trajectories using the one-way ANOVA and the Chi-square tests.

LCGA is a semi‐parametric technique used to identify distinct subgroups of individuals following a similar pattern of change over time on a given variable. In the current analysis, this approach identifies patterns of BMI, which determines mothers with similar behavioral trajectories. We applied the PROC TRAJ procedure extension for SAS to build group-based multi-trajectory models via a particular application of finite mixture modeling. Model selection was made in two steps. The number of trajectory groups was determined based on the Bayesian information criterion (BIC) and substantive significance^40^. In the second step, we tested the various shapes of each latent class to identify the pattern of change over time (linear, quadratic, or cubic). In all analyses, the minimal class sizes of at least 5% of the sample were considered. The models' goodness of fit was assessed using the average posterior probability (APP) of group membership ≥ 70% and that the odds of correct classification (OCC) ≥ 5 for each group and similarities between the estimated probability of the trajectory group and the proportion assigned to the group as suggested by Nagin^40^. Following the identification of the trajectory groups, each group was assigned a label regarding their pattern of BMI during the follow-up phases.

We examined the associations of each lifetime BMI trajectory group with cardiometabolic risk factors by logistic regression models. BMI trajectory group as a predictor was included in models, and the normal group was considered as a reference category. The first model was unadjusted; the second model was adjusted for age, education, employment, and marital status, and the third model was further adjusted for smoking status and physical activity. Also, in assessing the association between mother's trajectory groups and her offspring, sex was also considered in all mentioned models. Statistical analyses were performed using SAS (version 9.4; SAS Institute Inc) and IBM SPSS Statistics version 22. *P* < 0.05 was considered statistically significant.

### Ethics approval and consent to participate

This study was approved by the research ethics committee of the Research Institute for Endocrine Sciences (RIES), Shahid Beheshti University of Medical Sciences. All procedures were in accordance with the ethical standards of the institutional and/or national research committee and with the 1964 Helsinki declaration and its later amendments or comparable ethical standards.

### Informed consent

Informed consent was obtained from all individual participants included in the study. Before data collection, both children and parents were informed about the study procedure and its aims, and if the child and parent agreed to participate in the study, parents were asked to sign a written consent form.

## Results

### BMI trajectory patterns

**Figure 2:**
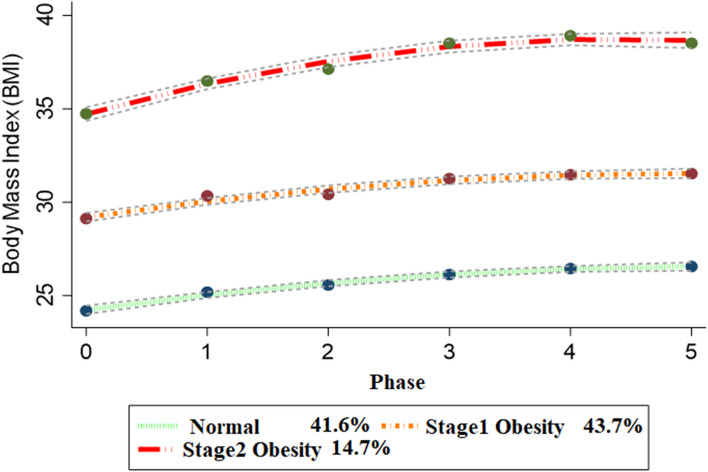
Women’s BMI trajectories. Dashed lines indicate the 95% confidence interval.

BMI of women for each predicted trajectory group across follow-up measurements was presented in Fig. 2. The LCGM identified three trajectory groups for BMI in 1356 mothers. Based on the goodness of fit criteria in Table 1-supplementary material, although four and five trajectory groups were statistically appropriate, a three-trajectory group was the best fitting model according to our basis hypothesis. The BIC for this model was − 18,208.71, and the APP ranged from 0.95 to 0.96. The OCC was 33.69, 26.70, and 169.72 for the first to third groups, respectively, and 41.6%, 43.70%, and 14.7% were grouped into class 1, class 2, and class 3, respectively. The three patterns were labeled as class 1/Normal, class 2/ Stage 1 obesity, class 3/ Stage 2 obesity. The normal trajectory pattern showed a steady normal BMI during baseline to the last follow-up assessments. The stage 1 obesity trajectory group showed a higher BMI increase during the follow-up period, whereas the BMI level was always within the stage 1 obesity range. The stage 2 obesity trajectory group was characterized by a steep rise in BMI over follow-ups, including those obese at baseline and remained in the same weight status in all follow-up measurements. Table 2-supplementary material shows BMI changes in each group over the follow-up period. As expected, in all trajectory groups, BMI increases through the follow-up period. The highest and lowest mean BMI were about 39 and 24 at the fourth and baseline measurements.

### Participants' characteristics based on the BMI trajectory groups in women

The characteristics of all participants (women, husbands, and offspring) based on women’s trajectories are shown in Table 1. The fathers ranged in age from 57 to 61 years old, and the average age of mothers varied between 51 to 55 years old in different groups, and the corresponding values for their offspring ranged from 27 to 28 years old. Comparing men’s characteristics in various women’s BMI trajectories showed a significant statistical difference for all examined variables except for dyslipidemia and smoking. Furthermore, apart from employment status, all women’s variables were significant among their BMI trajectory groups. In the last follow-up, compared to those in other BMI trajectory patterns, women in the stage 2 obesity trajectory group and their spouses were the oldest (*p* < 0.001). Most of them had a low level of education and were less physically active, and more smokers. In addition, hypertension and diabetes type 2 were more prevalent in steady obese mothers and their spouses. In terms of offspring, obesity, marital status, dyslipidemia, and age were statistically different among their mothers’ BMI trajectory groups. Children whose mothers were in the stage 2 obesity trajectory group had the highest prevalence of obesity (31.9%) and dyslipidemia (64.9%). In comparison, the corresponding values for children with mothers in the normal weight group were 9.1% and 52.3%, respectively. In addition, the prevalence of obesity and dyslipidemia were reported to be 18% and 58.7%, respectively, in children whose mothers were in the stage 1 obesity group.

**Table 1:** Descriptive statistics of whole family members across the trajectory pattern of women’s BMI.

	Husband				Women				Offspring			
	Normal	Stage1 Obesity	Stage2 Obesity	*P* value	Normal	Stage1 Obesity	Stage2 Obesity	*P* value	Normal	Stage1 Obesity	Stage2 Obesity	*P* value
**Sex**												0.31
Boy									222 (46.8)	268 (50.8)	84 (45.4)	
Girl									252 (53.2)	260 (49.2)	101 (54.6)	
**Age**	57.15 ± 7.75	59.98 ± 8.24	61.51 ± 8.73	< 0.001	51.43 ± 7.48	54.79 ± 7.75	55.74 ± 8.36	< 0.001	27.35 ± 4.61	28.61 ± 4.31	28.79 ± 3.89	< 0.001
**Education**				0.001				< 0.001				0.56
Primary	61 (15.2)	93 (22.9)	35 (23.6)		70 (15.5)	127 (28.1)	59 (41.3)		1 (0.2)	2 (0.4)	0	
Secondary	238 (59.2)	227 (55.8)	92 (62.2)		323 (71.3)	273 (60.4)	76 (53.1)		182 (38.4)	179 (33.9)	66 (35.7)	
Higher	103 (25.6)	87 (21.4)	21 (14.2)		60 (13.2)	52 (11.5)	8 (5.6)		291 (61.4)	347 (65.7)	119 (64.3)	
**Employment**				< 0.001				0.06				0.30
Employed	273 (67.9)	228 (56)	62 (46.6)		39 (8.6)	32 (7.1)	4 (2.8)		252 (53.2)	303 (57.4)	108 (58.4)	
Un-employed	129 (32.1)	179 (44)	71 (53.4)		414 (91.4)	420 (92.9)	139 (97.2)		222 (46.8)	225 (42.6)	77 (41.6)	
**Physical activity**	2423 ± 3616.8	1993. ± 2880.2	1707.6 ± 2394.1	0.04	1408.6 ± 1252	1423.8 ± 1218	1030.2 ± 1190.4	0.002	2322.9 ± 3662.4	2176.7 ± 3084.3	2367.7 ± 3491.5	0.71
**Smoking**				0.06								0.65
Smoker	103 (25.6)	78 (19.2)	21 (15.8)		14 (3.1)	6 (1.3)	4 (2.8)		78 (16.5)	91 (17.2)	36 (19.5)	
Non-smoker	299 (74.4)	329 (80.8)	112 (84.2)		439 (69.6)	446 (98.7)	139 (97.2)		396 (83.5)	437 (82.8)	149 (80.5)	
**HTN**				0.001				< 0.001				0.57
Yes	123 (30.6)	160 (39.3)	79 (53.4)		87 (19.2)	157 (34.7)	74 (51.7)		23 (4.9)	20 (3.8)	10 (5.4)	
No	279 (69.4)	247 (60.7)	69 (46.6)		366 (80.8)	295 (65.3)	69 (48.3)		451 (95.1)	508 (96.2)	175 (94.6)	
**DM**				0.001				< 0.001				0.20
Yes	80 (19.9)	104 (25.6)	43 (32.3)		63 (13.9)	122 (27)	60 (42)		9 (1.9)	13 (2.5)	8 (4.3)	
No	322 (80.1)	303 (74.4)	90 (67.7)		390 (86.1)	330 (73)	83 (58)		465 (98.1)	515 (97.5)	177 (95.7)	
**Dyslipidaemia**				0.46				< 0.001				0.009
Yes	322 (80.1)	327 (80.3)	125 (84.5)		357 (78.8)	394 (87.2)	130 (90.9)		248 (52.3)	310 (58.7)	120 (64.9)	
No	80 (19.9)	80 (19.7)	23 (15.5)		96 (21.2)	58 (12.8)	13 (9.1)		226 (47.7)	218 (41.3)	65 (35.1)	
**Obesity**				0.34								< 0.001
Yes	96 (23.9)	103 (25.3)	42 (31.6)						43 (9.1)	95 (18)	59 (31.9)	
No	306 (76.1)	304 (74.7)	91 (68.4)						431 (90.9)	433 (82)	126 (68.1)	
**Marital**												0.001
Single									260 (54.9)	254 (48.1)	71 (38.4)	
Married									214 (45.1)	274 (51.9)	114 (61.6)	
**Baseline characteristic**												
BMI	26.03 ± 3.78	26.53 ± 3.74	26.74 ± 3.31	0.033					24.37 ± 4.32	26.42 ± 4.64	28.07 ± 5.55	< 0.001
SBP	116.43 ± 14.90	120.09 ± 16.92	119.15 ± 15.83	0.001	111.40 ± 13.67	116.10 ± 14.93	121.21 ± 15.98	< 0.001	103.66 ± 11.29	104.80 ± 11.29	104.94 ± 11.89	0.044
DBP	77.94 ± 10.48	79.17 ± 10.92	78.54 ± 9.81	0.178	74.56 ± 9.42	78.31 ± 9.71	81.58 ± 9.92	< 0.001	70.60 ± 9.60	70.43 ± 9.56	70.87 ± 9.76	0.867
FPG	93.27 ± 17.82	98.41 ± 28.80	100.71 ± 31.85	< 0.001	91.36 ± 24.32	94.08 ± 21.45	93.74 ± 21.04	0.112	86.67 ± 8.59	87.37 ± 8.25	88.10 ± 8.04	0.008
TG	194.84 ± 134.93	199.53 ± 136.41	206.15 ± 115.03	0.610	131.89 ± 76.21	166.94 ± 94.27	179.12 ± 96.07	< 0.001	98.68 ± 48.19	105.25 ± 55.20	107.20 ± 53.61	0.227
TC	212.34 ± 43.99	210.00 ± 38.16	213.86 ± 42.19	0.480	197.58 ± 39.98	212.22 ± 42.43	214.30 ± 40.67	< 0.001	167.92 ± 30.47	168.91 ± 29.62	171.47 ± 37.83	0.005
HDL-C	37.64 ± 8.74	37.78 ± 9.76	38.36 ± 8.61	0.672	45.81 ± 10.54	43.15 ± 11.29	44.12 ± 11.98	< 0.001	45.02 ± 10.42	43.99 ± 10.37	44.81 ± 11.46	0.108

Values are expressed as mean ± SD for continuous variables and n (%) for categorical Variables.

BMI, body mass index; SBP, Systolic *blood* pressure, DBP, diastolic *blood* pressure; FPG, *Fasting Plasma Glucose;* TG, Triglycerides; TC, Total Cholesterol; HDL-C, High-density lipoprotein cholesterol.

### Women's BMI trajectory patterns and familial cardiovascular risk factors

As presented in Table 2, in all models, compared to women's in the normal trajectory group, those in stage 1 and 2 of obesity trajectory groups had higher odds ratios for HTN, DM, and dyslipidemia (p < 0.05). The second model showed a higher rate of DM in men with obese (stage 2) spouses (OR 1.56; 95% CI 1.01–2.39) as well as a higher rate of HTN 1.54 (95% CI 1.05–2.25) even after adjusting for potential confounders. The odds ratio for DM remained significant after adjusting behavioral factors in model 3 (adjusted OR 1.55; 95% CI 1.00–2.44). In the second and third models, the odds of men's obesity were higher in their obese (stage 2) spouses compared to normal weight ones, and the odds ratios were (OR 1.65; 95% CI 1.09–2.51) and (OR 1.70; 95% CI 1.10–2.62) respectively. In the full adjusted model, offspring of obese (stage 1 and 2) mothers were more likely to be obese than those whose mothers were normal weight. The odds ratios were (OR 2.39; 95% CI 1.67–3.44) and (OR 4.81; 95% CI 3.16–7.34) respectively. The corresponding values for dyslipidemia in offspring with obese (stage 2) mothers were 1.42 (95% CI 1.01–1.98) and 1.39 (95% CI 1.00–1.94) in the second and third models, respectively. No significant associations were found between the trajectory groups of mothers and their offspring’s outcome, including HTN and DM.

**Table 2:** Association of women’s trajectories of BMI with the whole family outcomes.

	Women			Husband				Offspring			
	HTN	DM	Dyslipidaemia	HTN	DM	Dyslipidaemia	Obesity	HTN	DM	Dyslipidaemia	Obesity
	OR (95%CI)	OR (95%CI)	OR (95%CI)	OR (95%CI)	OR (95%CI)	OR (95%CI)	OR (95%CI)	OR (95%CI)	OR (95%CI)	OR (95%CI)	OR (95%CI)
**Model 1**											
Normal	Ref	Ref	Ref	Ref	Ref	Ref	Ref	Ref	Ref	Ref	Ref
Stage1 Obesity	2.08 (1.56–2.76)	2.24 (1.60–3.13)	1.80 (1.28–2.53)	2.13 (1.60–2.84)	1.42 (1.03–1.95)	0.97 (0.71–1.33)	1.10 (0.82–1.47)	0.98 (0.57–1.70)	1.39 (0.6–3.26)	1.27 (1.01–1.59)	2.18 (1.55–3.07)
Stage2 Obesity	4.03 (2.76–5.89)	4.44 (2.91–6.77)	2.39 (1.36–4.19)	3.58 (2.45–5.23)	1.89 (1.24–2.86)	0.86 (0.56–1.32)	1.33 (0.90–1.96)	1.14 (0.55–2.36)	2.33 (0.88–6.13)	1.61 (1.16–2.23)	4.15 (2.82–6.10)
**Model 2**											
Normal	Ref	Ref	Ref	Ref	Ref	Ref	Ref	Ref	Ref	Ref	Ref
Stage1 Obesity	1.66 (1.22–2.26)	1.78 (1.25–2.52)	1.58 (1.11–2.24)	1.23 (0.93–1.62)	1.25 (0.90–1.73)	0.99 (0.71–1.36)	1.24 (0.91–1.67)	0.93 (0.53–1.63)	1.26 (0.54–2.97)	1.19 (0.94–1.50)	2.37 (1.65–3.40)
Stage2 Obesity	3.24 (2.15–4.88)	3.54 (2.26–5.55)	2.04 (1.15–3.62)	1.54 (1.05–2.25)	1.56 (1.01–2.39)	0.88 (0.57–1.37)	1.65 (1.09–2.51)	1.06 (0.50–2.21)	2.10 (0.79–5.60)	1.42 (1.01–1.98)	4.85 (3.18–7.39)
**Model 3**											
Normal	Ref	Ref	Ref	Ref	Ref	Ref	Ref	Ref	Ref	Ref	Ref
Stage1 Obesity	1.65 (1.21–2.24)	1.85 (1.30–2.64)	1.56 (1.10–2.22)	1.21 (0.91–1.61)	1.20 (0.85–1.68)	1.01 (0.72–1.41)	1.18 (0.86–1.62)	0.92 (0.52–1.61)	1.25 (0.53–2.94)	1.19 (0.94–1.51)	2.39 (1.67–3.44)
Stage2 Obesity	3.19 (2.11–4.83)	3.59 (2.28–5.64)	1.98 (1.11–3.52)	1.41 (0.94–2.11)	1.55 (1.00–2.44)	0.88 (0.55–1.41)	1.70 (1.10–2.62)	1.02 (0.49–2.15)	2.09 (0.78–5.57)	1.39 (1.00–1.94)	4.81 (3.16–7.34)

Model 1: unadjusted OR (95% CI).

Model 2: adjusted for age, education, employment, and marital status.

Model 3: adjusted for age, education, employment, marital status, smoking, and physical activity.

*In offspring, Model 2 and 3 are adjusted for sex in addition to the covariates mentioned above.

## Discussion

The current study aimed to evaluate the trajectory patterns of women’s BMI and to determine the association between these patterns with cardiovascular risk factors at both individual and familial levels. Using six measurements of women's BMI during a 20 years follow-up period, three rising patterns of BMI were identified, including normal, stage 1 obesity, and stage 2 obesity. Our findings indicated that women in obese trajectories were more likely to have HTN, DM, and dyslipidemia. In addition, men with obese (stage 2) spouses showed a higher rate of HTN and DM. In terms of the mother–offspring relationship, the odds of obesity in offspring with obese (stage 1 and 2) mothers were higher than those whose mothers were normal weight.

Few studies investigated developmental trajectories of BMI in particular among women. In this novel longitudinal study, we assessed trajectory patterns of BMI among Iranian women. The model classified individuals in three rising BMI patterns. The current findings were consistent with world health organization (WHO) cut-off points for BMI categorization^41^. Although there is no comparable data, we compared with other studies on BMI patterns in specific life periods. A study conducted among the Chinese population aged 6–60 years identified a continuous increase of BMI in all groups of BMI^42^. Some studies indicated less variability in the BMI trajectories in midlife in the total population and women^43–45^, whereas another study reported different patterns during middle age^46^. A survey conducted among Australian women showed significantly three distinct BMI patterns, similar to our findings^47^. There is only one study in Iran that determined two patterns of BMI; however, their sample included adolescents^28^.

In addition, we linked the trajectory pattern of women’s BMI to several cardiovascular risk factors, including high blood pressure, high blood glucose, obesity, and dyslipidemia. Our findings indicated that women in both obesity (stage 1 and 2) trajectories were more likely to have HTN, DM, and dyslipidemia, suggesting the importance of early intervention. Previous studies have illustrated that an elevated BMI in women contributes to a high risk for DM, HTN, and high-risk HDL cholesterol, consistent with our results^48–50^. In contrast, another study indicated that a rising trend of BMI was not associated with high blood pressure. This finding may have been due to other related factors with blood pressure, such as smoking^51^. Compared with the current study, different statistical methods applied among these studies and the limited time points would also be considered. Additionally, differences among populations such as ethnicity, culture, and socioeconomic status (SES) may also explain this inconsistency^52^.

Our study showed that men with obese spouses showed a higher rate of HTN and DM in terms of spousal association. There was no comparable data regarding the effect of one spouse’s weight trajectories on cardio-metabolic risk factors in the other spouse. However, in line with our results, some cross-sectional studies considered the spousal concordance in chronic diseases. Studies showed that husbands' health status is influenced by their wives who get chronic diseases^53^, and husbands' weight status were linked to their spouses’ DM. Moreover, another study showed this association in the other direction in which the weight gain in wives increased their husbands' risk of type 2 diabetes^21^. The reasons may be explained by the predominant role of wives as caregivers in the families^54^. Regarding lifestyles and health management, husbands may be more dependent on their spouses. Women with obese spouses are more likely to have chronic diseases such as obesity, HTN, and DM^55^.

Regarding the association between women’s BMI and CVD risk factors in their offspring, the current study reported that mothers with high BMI values are predicted to have obese children, consistent with previous studies^56^. Moreover, some cross-sectional studies in Iran and other countries reported that children with obese parents had significantly higher odds of obesity and elevated BP^18,57,58^. This association is more attributed to the mother–offspring relationship^59^. This may be due to genetic/epigenetic factors, shared family environment, and parents' health-related attitudes, particularly mothers, because of their leading roles in the family, which influence their children's behaviors^60^. Although some studies reported the association between parental obesity and HTN^61^, DM^62^ of their offspring, an investigation did not find any significant association which was in line with the current results^62,63^.

This study has several strengths. This is the first report in the world, which addressed the association of BMI trajectories with cardiovascular risk factors at both individual and familial levels. The current study was conducted among the Iranian population with a relatively large sample and a long follow-up period. From an analytical perspective, LCGA was applied as a precise longitudinal method to characterize distinct BMI trajectory patterns. However, several limitations of this study need to be considered. First, the sample was limited to a metropolitan city and cannot be generalized to a rural population. Secondly, some potential predictors of cardiovascular risk factors such as genetic susceptibility, diet data, and environmental conditions such as air pollution^64,65^ were not available in the current study.

## Conclusion

In conclusion, to our knowledge, for the first time in a relatively large community-based sample of Iranian families, our data identified three developmental trajectories of women's BMI and showed significant associations between these trajectories with obesity, dyslipidemia, HTN, and DM in their spouses and children. These findings indicated the critical role of a shared environment in determining cardiovascular risk factors. Accordingly, family-based prevention strategies should be considered for controlling cardiovascular risk factors in whole family members.

## Supplementary Information

Supplementary Tables.

## Data Availability

The datasets used and/or analysed during the current study are available from the corresponding author on reasonable request.
