# Supplementary material for: Predictive value of women’s weight trajectories in determining familial cardiovascular disorders: a family-based longitudinal study
Source: Sci Rep. 2021 Aug 27;11:17317. doi: 10.1038/s41598-021-96154-5 (PMC8397790; doi:10.1038/s41598-021-96154-5)
Supplement: Supplementary file 1 — Supplementary Tables. [file 41598_2021_96154_MOESM1_ESM.docx]

**Supplementary material**

**Table 1.** Model fit characteristics for trajectory model analysis.

| **Trajectory group** | **N** | **Estimated (SE)** | **Assigned**  **(%)** | **APP (%)** | **OCC** | **BIC**  **(n=7213)** | **BIC (n=1356)** |
| --- | --- | --- | --- | --- | --- | --- | --- |
| **2-group model** |  |  |  |  |  | -19288.23 | -19281.54 |
| 1 | 972 | 71.62 (1.37) | 71.68 | 0.98 | 22.79 |  |  |
| 2 | 384 | 28.37 (1.37) | 28.31 | 0.95 | 58.76 |  |  |
| **3-group model** |  |  |  |  |  | -18218.73 | -18208.71 |
| 1 | 566 | 41.55 (1.56) | 41.74 | 0.96 | 33.69 |  |  |
| 2 | 591 | 43.71 (1.51) | 43.58 | 0.95 | 26.70 |  |  |
| 3 | 199 | 14.73 (1.06) | 14.67 | 0.96 | 169.72 |  |  |
| **4-group model** |  |  |  |  |  | -17556.89 | -17543.52 |
| 1 | 435 | 32.11 (1.45) | 32.07 | 0.95 | 47.94 |  |  |
| 2 | 578 | 42.46 (1.50) | 42.62 | 0.95 | 25.75 |  |  |
| 3 | 270 | 19.97 (1.16) | 19.91 | 0.95 | 93.99 |  |  |
| 4 | 73 | 5.45 (0.65) | 5.38 | 0.97 | 657.81 |  |  |
| **5-group model** |  |  |  |  |  | -17152.55 | -17135.83 |
| 1 | 128 | 9.81 (0.99) | 9.43 | 0.94 | 162.90 |  |  |
| 2 | 467 | 33.84 (1.46) | 34.43 | 0.93 | 25.44 |  |  |
| 3 | 447 | 33.22 (1.45) | 32.96 | 0.94 | 34.81 |  |  |
| 4 | 246 | 18.04 (1.10) | 18.14 | 0.95 | 104.09 |  |  |
| 5 | 68 | 5.07 (0.64) | 5.01 | 0.97 | 808.78 |  |  |
| **6-group model** |  |  |  |  |  | -16941.10 | -16921.05 |
| 1 | 126 | 9.43 (0.93) | 9.29 | 0.94 | 160.90 |  |  |
| 2 | 445 | 32.55 (1.43) | 32.81 | 0.93 | 30.97 |  |  |
| 3 | 440 | 32.20 (1.43) | 32.44 | 0.93 | 30.02 |  |  |
| 4 | 220 | 16.40 (1.11) | 16.22 | 0.94 | 88.51 |  |  |
| 5 | 87 | 6.51 (0.73) | 6.41 | 0.93 | 218.31 |  |  |
| 6 | 38 | 2.88 (0.64) | 2.80 | 0.96 | 899.35 |  |  |

**Table 2.** Women’s BMI over follow-ups.

|  |  | **Mean BMI** |  |
| --- | --- | --- | --- |
|  | **Normal** | **Stage 1 obesity** | **Stage 2 obesity** |
| **Baseline** | 24.19±2.50 | 29.12±2.34 | 34.66±3.14 |
| **Follow-up 1** | 25.25±2.23 | 30.35±2.16 | 36.47±2.79 |
| **Follow-up 2** | 25.59±2.12 | 30.40±2.01 | 36.87±3.39 |
| **Follow-up 3** | 26.13±2.20 | 31.21±2.22 | 38.34±3.85 |
| **Follow-up 4** | 26.42±2.21 | 31.48±2.34 | 38.80±3.58 |
| **Follow-up 5** | 26.55±2.28 | 31.60±2.40 | 38.69±4.04 |
